# Cytosolic DNA sensing protein pathway is activated in human hearts with dilated cardiomyopathy

**DOI:** 10.20517/jca.2023.20

**Published:** 2023-07-10

**Authors:** Leila Rouhi, Sirisha M. Cheedipudi, Benjamin Cathcart, Priyatansh Gurha, Ali J. Marian

**Affiliations:** Center for Cardiovascular Genetics, Institute of Molecular Medicine, The University of Texas Health Science Center. Houston TX 77030, USA.

**Keywords:** DNA damage, double-stranded DNA breaks, cardiomyopathy, heart failure

## Abstract

**Introduction:**

The genome is constantly exposed to numerous stressors, which induce DNA lesions, including double-stranded DNA breaks (DSBs). DSBs are the most dangerous, as they induce genomic instability. In response to DNA damage, the cell activates nuclear DNA damage response (DDR) and the cytosolic DNA sensing protein (CDSP) pathways, the latter upon release of the DSBs to the cytosol. The CDSP pathway activates NFκB and IRF3, which induce the expression of the pro-inflammatory genes. There is scant data on the activation of the CDSP pathway in human hearts with dilated cardiomyopathy (DCM).

**Aim:**

We aimed to determine expression levels of selected components of the CDSP pathway in human hearts with DCM.

**Methods:**

The DNA strand breaks were detected by the single-cell gel electrophoresis or the comet assay and expression of selected proteins by immunoblotting. Transcript levels were quantified in the RNA-Seq data.

**Results:**

Single-cell gel electrophoresis showed an approximately 2-fold increase in the number of COMET cells in the DCM hearts. Immunoblotting showed increased levels of cyclic GMP-AMP synthase (CGAS), the canonical CDSP; TANK-binding kinase 1 (TBK1), an intermediary kinase in the pathway; and RELB, P52, and P50 components of the NFκB pathway in human heart samples from patients with DCM. Likewise, transcript levels of over 2 dozen genes involved in inflammatory responses were increased.

**Conclusions:**

The findings provide the first set of evidence for the activation of the CDSP pathway in human hearts with DCM. The data in conjunction with the previous evidence of activation of the DDR pathway implicate the DSBs in the pathogenesis of human DCM.

## Graphical Abstract

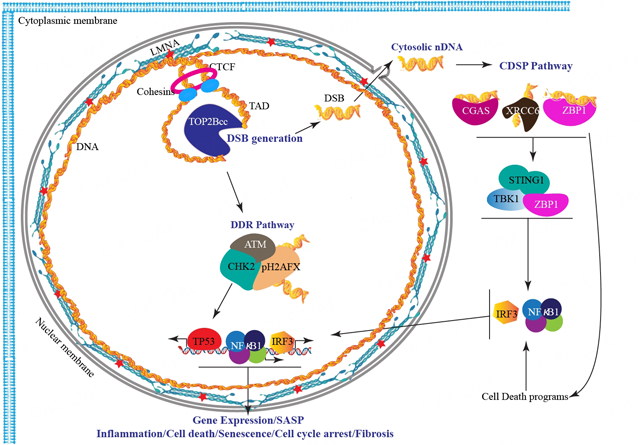

Various stressors cause DNA lesions, including double-stranded DNA breaks (DSBs). DSBs activate the nuclear DNA Damage Response (DDR) pathway, comprised of H2AFX, ATM, CHK2, and TP53. The latter induces expression of genes involved in cell cycle arrest, cell death, senescence, and DNA repair among others.

DSBs are also released into the cytosol where the DNA activated the cytosolic DNA sensing proteins, such as CGAS, leading to the activation of a cascade of proteins that merge into activating NFkB and IRF3. These transcription factors induce the expression of pro-inflammatory genes and senescence-associated secretory phenotype (SASP).

## INTRODUCTION

The integrity of the genome is essential for survival and its instability is a hallmark of aging^[1]^. The nuclear genome, however, is incessantly exposed to damaging external and internal stressors, such as ionizing radiation and replication/transcription stress^[2,3]^. The stressors induce chemical and structural lesions in the DNA, including strand breaks at an estimated rate of 105 lesions in a mammalian cell per day^[4]^. Double-stranded DNA breaks (DSBs), which are the most dangerous DNA lesions are less common and occur at an estimated rate of 50 DSBs per mammalian cell per day^[5,6]^. To maintain genomic stability, the DSBs are quickly repaired by a set of repair proteins that are recruited to the break sites^[6]^. In non-replicating cells such as cardiac myocytes, DSBs are mainly repaired by non-homologous end joining (NHEJ), which is error-prone, as opposed to homology-directed repair (HDR)^[6]^. NHEJ introduces insertion-deletion (indels) mutations with potentially detrimental effects on gene function^[7,8]^.

Under cell stress, the balance between the generation and repair of DSBs shifts toward the accumulation of DSBs in the nucleus^[6]^. Within the nucleus, the unrepaired DSBs activate the DNA damage response (DDR) pathway involving the ataxia-telangiectasia mutated (ATM)-tumor protein 53 (TP53) axis, which leads to cell cycle arrest, inflammation, senescence, and cell death^[5,6]^. A subset of the DSBs is also released to the cytosol, which is then sensed by the cytosolic DNA-sensing proteins (CDSPs), such as cyclic GMP-AMP synthase (CGAS)^[9,10]^. CGAS activates, through several intermediary molecules, the nuclear factor kappa B (NFκB) and interferon regulatory factor 3 (IRF3) pathways, which in turn induce expression of the pro-inflammatory genes^[9,10]^.

We have previously shown the nuclear DDR and the CDSP pathways are activated in the mouse models of dilated cardiomyopathy (DCM) caused by deficiency of the nuclear envelope proteins, including LMNA and TMEM43, or the cardiac-myocyte restricted expression of the mutant LMNA^D300N^ protein, the latter is known to cause non-syndromic cardiac progeria in humans^[11–14]^. In addition, we have shown that the deletion of genes encoding CGAS and TP53, representing the CDSP and DDR pathways, respectively, attenuates the phenotype in mouse models of DCM^[13,15]^. The genetic blockade of the CDSP or DDR pathway prolongs survival, improves cardiac function, reduces myocardial fibrosis, and attenuates cell death^[13,15]^. The salubrious effects of blocking the DDR and CDSP pathways demonstrated the clinical significance of the activation of these pathways and their pathogenic roles in cardiomyopathies.

Despite the data in the mouse models, there is scant evidence of activation of the DDR and CDSP pathways in human hearts from patients with DCM. We have previously shown upregulation of the components of the DDR pathway, namely ATM, H2A histone family, member X (H2AFX), and TP53 in the human hearts with DCM^[13,16]^. However, whether the CDSP pathway, which indicates the release of the DNA into the cytosol, is also activated in human hearts from patients with DCM remains unknown.

## METHODS

### Single-cell gel electrophoresis:

To detect DNA strand beaks, we performed single-cell gel electrophoresis or Comet assay to separate the DNA fragments from the intact DNA using a commercial kit (OxiSelect^™^ Comet Assay, Cell Biolab Inc. Catalogue number STA-351)^[17]^. In brief, 2mg aliquots of left ventricular heart tissues from four control donor hearts, not used for transplantations, and four explanted hearts of patients with primary DCM (Caucasians, 3 male and 1 female, mean age: 51 ± 12) were minced into small pieces in a PBS containing 20mM EDTA followed by centrifugation at 1200 rpm for 8 min^[13,18]^. All samples were from the left ventricular tissues and were transmural, however, the exact segment(s) of the left ventricles that were excised and used in these experiments was unknown. The cell pellet was mixed with the comet agarose solution and transferred onto the OxiSelect^™^ Comet Slide. Slides were incubated in 25ml lysis buffer for 45 min followed by incubation in the alkaline solution for 30 min at 4 °C in the dark. The samples are subjected to electrophoresis on a horizontal gel electrophoresis chamber at 35 V for 30 min. The slides are washed twice in prechilled deionized water three times followed by treatment with 70% ethanol for 5 min. The samples were dried and stained with Vista Green DNA dye (1:10,000 in TE buffer) for 15 min at room temperature. The DNA damage was identified by counting the number of nuclei showing a fluorescence signal emitting from the nuclei as a comet tail.

### Immunoblotting:

Expression levels of selected CDSPs were analyzed by immunoblotting, as published^[13,15]^. In brief, aliquots of 30 to 50 μg of total cardiac protein extracts were loaded onto polyacrylamide gels, electrophoresed, and transferred to nitrocellulose membranes. The membranes were probed with antibodies against the target proteins. The expression levels of CGAS, X-ray repair cross-complementing protein 6 (XRCC6, a.k.a. Ku70), XRCC5 (a.k.a. Ku80), stimulator of interferon genes 1 (STING1 a.k.a. TMEM173), phospho-STING1 (serine residue 365), and TANK-binding kinase 1 (TBK1) were detected using the following antibodies: (CGAS: D1D3G, Rabbit mAb, Cell Signaling, Cat# 15102S; XRCC6: D10A7, Rabbit mAb, Cell Signaling, Cat# 4588S; XRCC5: C48E7, Rabbit mAb, Cell signaling, Cat# 2180S; STING1: Rabbit PolyAb, Protein Tech, Cat# 19851-1-AP; p-STING1: S365, D8F4W, Rabbit mAb, Cell Signaling, 72971S0; TBK1: D1B4, Rabbit mAb, Cell Signaling, Cat# 3504S).

The expression levels of downstream effectors of the CDSP pathway, namely transcription factor RelB (RELB), p52, p50, p65, IRF3, and pIRF3 (serine 396) were analyzed by immunoblotting using specific antibodies [RelB: D7D7W, Rabbit mAb, Cell signaling, Cat# 10544S; p100/p52: Rabbit Ab, Cell signaling, Cat# 4882S; p105/p50: D4P4D, Rabbit mAb, Cell Signaling, Cat# 13586S; p65: D14E12, XP(R) Rabbit mAb, Cell Signaling, Cat# 8242S; IRF-3: D83B9, Rabbit mAb, Cell signaling, Cat# 4302S and p-IRF-3: S396, 4D4G, Rabbit mAb, Cell Signaling, Cat# 4947S].

### Transcripts analysis:

Transcript levels of over two dozen genes involved in the inflammatory pathways were quantified in the RNA-sequencing data from the DCM hearts and presented as relative fold changes compared to the control hearts^[13,18]^.

## RESULTS

### DNA strand breaks:

**Figure 1.**
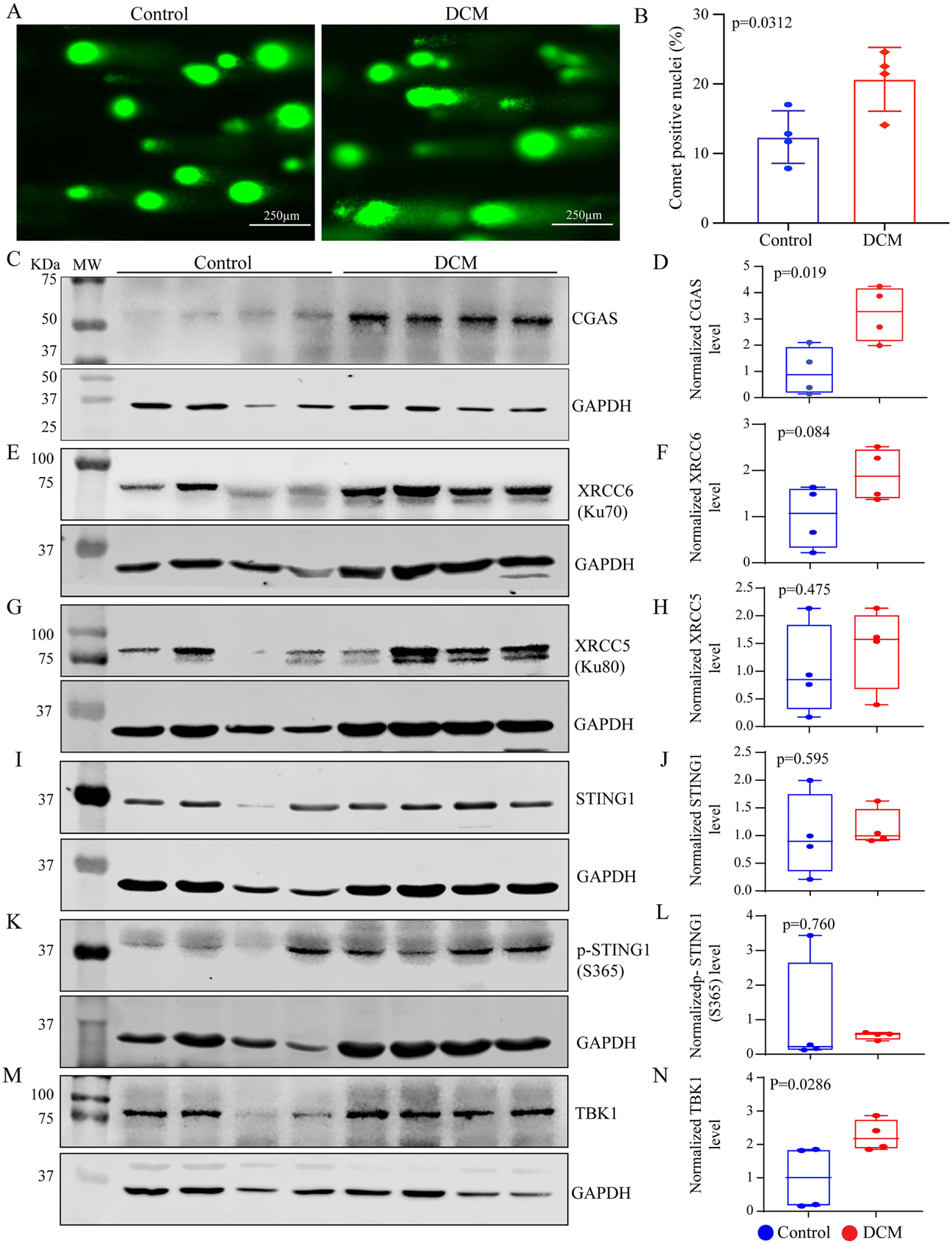
DNA strand breaks (COMET assay) and expression of the cytosolic DNA sensing proteins (CDSPs). (A) Immunofluorescence panels showing COMET cells, identified as cells showing fluorescence signal emitting from the nuclei as a comet tail, in the control and dilated cardiomyopathy (DCM) heart samples; (B) Quantitative data on the percentage of cells showing COMET cells (nuclei tails in green); (C-N) Immunoblots showing expression levels of selected CDSPs, as labeled on each blot, along with their quantitative data. The expression level of each protein is normalized to its corresponding GAPDH level and shown as relative levels. Box and whisker plots are shown and include the individual data points, the median, and the minimum and maximum values. All data followed the Gaussian distribution, as analyzed by the Shapiro-Wilk normality test. The differences between the two groups were compared by unpaired *t*-test.

The number of comet-positive nuclei counted in at least 2,000 cells per group, which showed an approximately 2-fold increase in the primary DCM heart samples as compared to control donor hearts [Figure 1A and B].

### Expression levels of selected CDSPs:

The expression level of CGAS, a bona fide cytosolic DNA sensor protein was markedly increased in the human heart samples from patients with primary DCM [Figure 1C and D]. The expression level of XRCC6 (Ku70), which has a dual function of sensing the cytosolic DNA as well as repair of the DSBs, was also increased [Figure 1E and F]. In contrast expression level of XRCC5 (Ku80), which is a repair protein with no known cytosolic DNA sensing function, was unchanged [Figure 1G and H]. Likewise, levels of STING1 (TMEM173) and phospho-STING1 (serine residue 365), which are multi-functional proteins in the CDSP pathway, were also unchanged [Figure 1I–L]. In contrast, the TBK1 protein level, which is a downstream effector of the CDSP pathway, was increased in the primary DCM hearts [Figure 1M and N].

**Figure 2.**
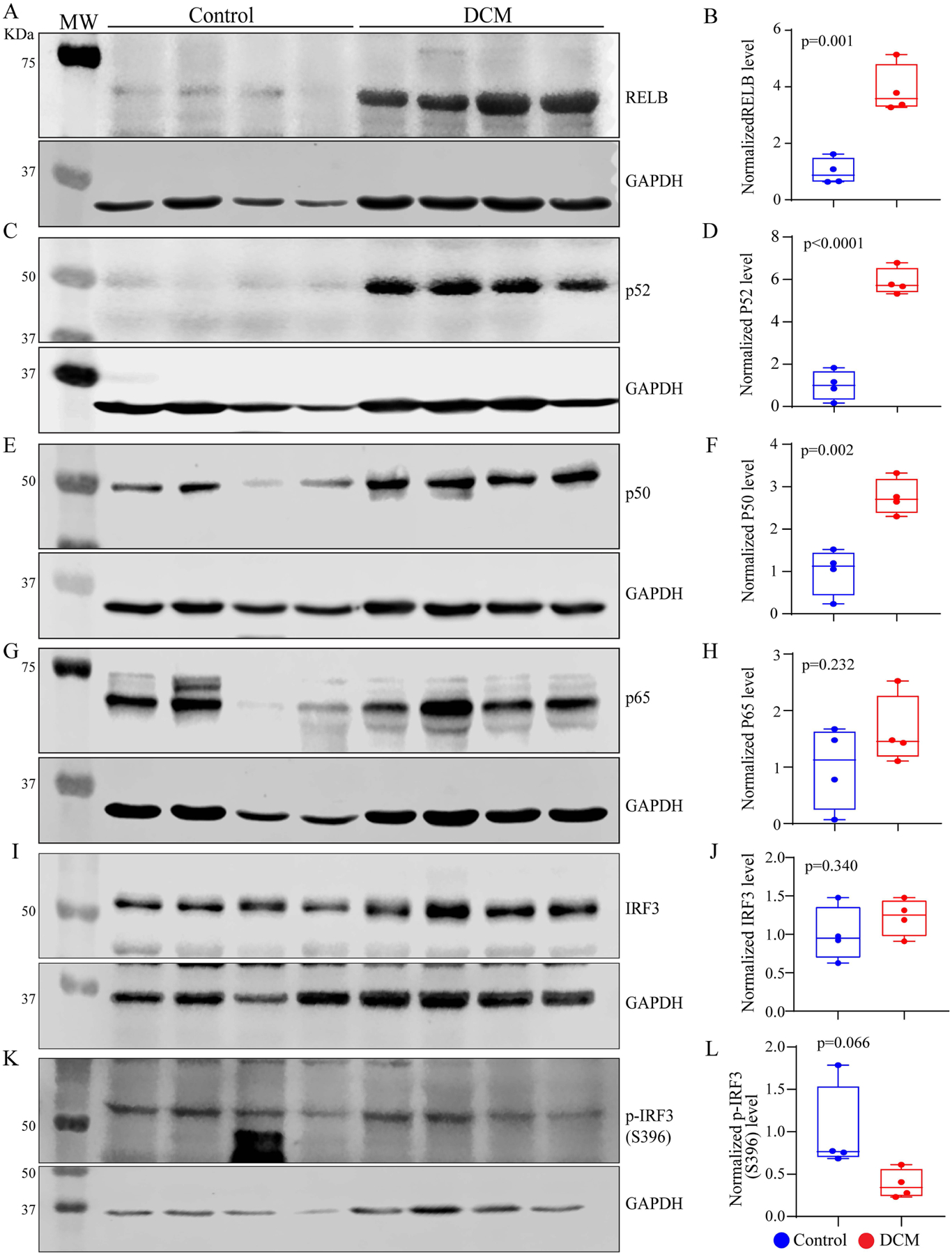
Expression of the effectors of the CDSP pathway. (A-L) Immunoblots showing expression levels of the components of the NFκB and IRF3 pathways in the control and human hearts with dilated cardiomyopathy (DCM), along with the corresponding quantitative data. Statistical analysis was as in Figure 1.

Given that activation of the CDSP pathway includes activation of NFκB and IRF3 transcription factors, levels of the protein components of these pathways were analyzed in the control and primary DCM hearts. The expression levels of RELB and p52, which dimerize to translocate into the nucleus and activate expression of the non-canonical NFκB target genes, were markedly increased in the primary DCM hearts [Figure 2A–D]. Likewise, the level of p50 protein, which is a component of the canonical NFκB, was increased, whereas the p65 level was unchanged [Figure 2E–H]. To assess the role of the innate immune response against the cytosolic DNA, protein levels of IRF3 and phospho-IRF3 (Serine 396) were analyzed by immunoblotting and were unchanged [Figure 2I–L].

### Transcript levels of genes involved in the inflammatory pathways:

**Figure 3.**
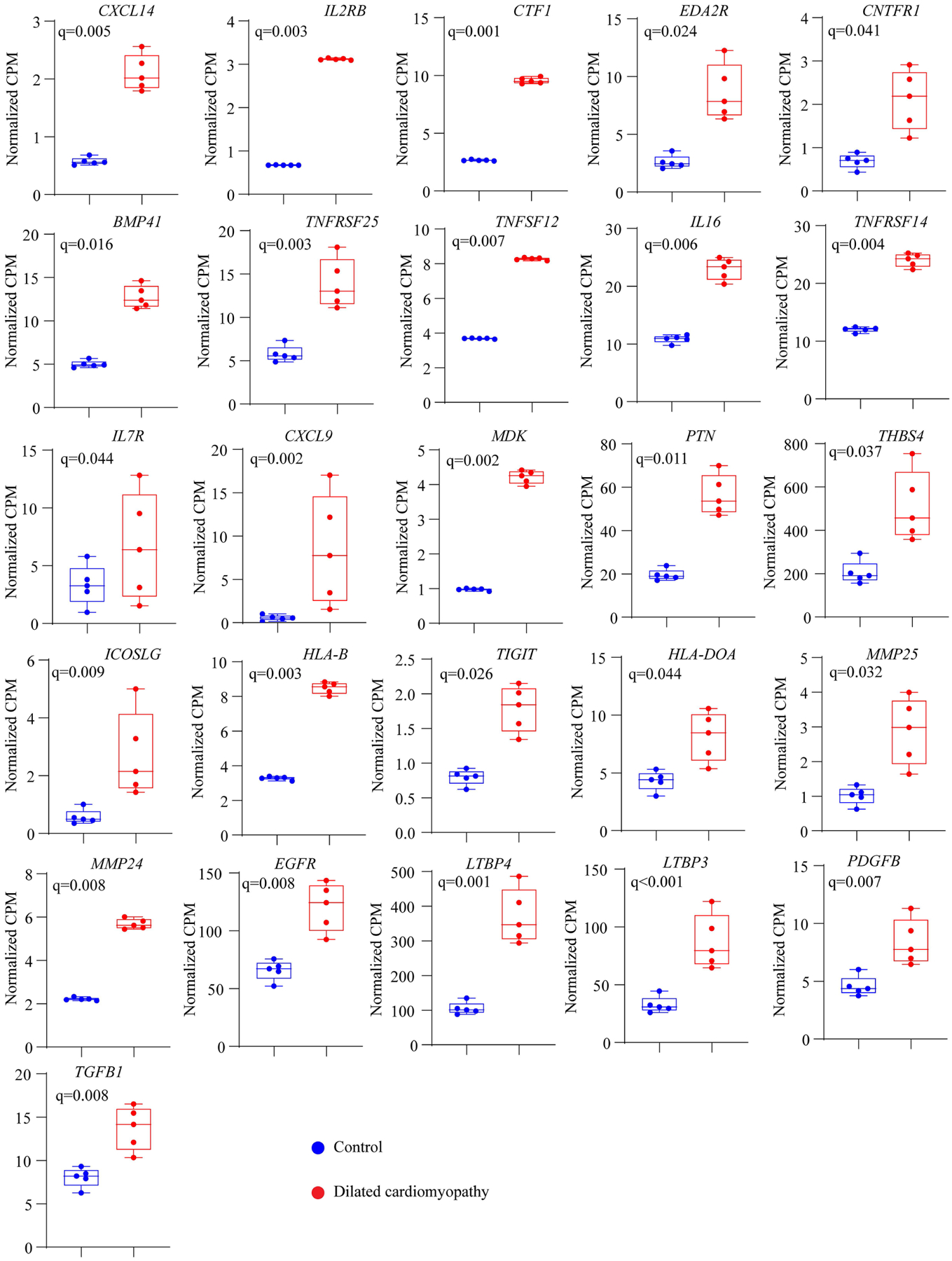
Box and whisker plots showing transcript levels of over a dozen genes involved in the inflammatory pathways. The normalized transcript levels, shown as count per million (CPM), were compared between the two groups as described in Figure 1.

To determine whether increased protein levels of the components of the NFκB pathway was associated with the upregulation of expression of genes involved in inflammatory pathways, transcript levels of over two dozen genes were analyzed in the RNA-sequencing data^[13,18]^. The data show increased transcript levels of 26 protein-coding genes involved in the inflammatory pathways, including members of the cytokine and tumor necrosis factor α family [Figure 3].

## DISCUSSION

To our knowledge, this is the first documentation of increased expression levels of the protein components of the CDSP pathway, namely CGAS, TBK1, RELB, P52, and P50 in the human heart samples from patients with heart failure due to DCM. These findings by showing the upregulation of expression of the CDSPs, including the components of the NFκB pathway, complement the previous data on the activation of the nuclear DDR pathway in human heart tissues from patients with DCM. The findings in the human heart samples, being devoid of the genetic manipulations, also give credence to the findings in the model organisms, which are subject to the experimental conditions, including the expression of Cre recombinase, known to cut the mammalian genome promiscuously at the pseudo-LoxP sites^[19,20]^. Collectively, the data implicate increased DSBs and activation of the DDR and CDSP pathways in the pathogenesis of the DCM and set the stage for further delineation of the pathogenic role of these pathways in human primary DCM. Given the salubrious effects of targeting the CDSP pathway in mouse models of DCM, the findings raise the prospects for targeting the activated CDSP pathways for the prevention and attenuation of the phenotype in human primary DCM. Given that cell stress, including transcriptional stress, is common to various forms of cardiovascular pathology, one may speculate that increased DSBs and activation of DDR and the CDSP pathways are pervasive and ubiquitous features of cardiovascular diseases.

The study has several limitations, including the small sample size of the cases and controls as well as its descriptive, albeit informative, nature. Thus, confirmation of the findings in larger sample-size studies would be valuable. The findings might be subject to data ascertainment and analysis bias, as immunoblotting was performed with the knowledge of the groups. However, to reduce the potential bias, an equal amount of each left ventricular tissue was used in these experiments and the loading controls were included and used for data normalization. The findings also might be influenced by the presence of regional differences in the myocardial gene expression and cellular composition, as the exact segment(s) of the left ventricles that were excised and used in these experiments were not defined at the time of sample collection^[21,22]^. In addition, the samples were stored for several years at −130 degrees, which might affect the integrity of the transcripts and proteins, particularly the phosphorylated forms of the selected proteins. Likewise, the DNA lesions might have occurred *ex vivo* and during sample storage. However, the inclusion of a control group and evidence of increased DSBs in the primary DCM samples, as opposed to controls, partially alleviates this concern. In addition, transcriptional activities of the canonical and non-canonical NF κB components were detected only at the transcript levels for a selected number of the target genes involved in inflammatory pathways. Moreover, the origin of the cytosolic DNA that activated the CGAS pathway could be not determined. It is plausible that both nuclear DNA and mitochondrial DNA, upon release into the cytosol, could activate the CGAS pathway^[23]^. Considering our previous data showing activation of the nuclear component of the DDR pathway, namely the H2AFX/ATM/TP53 pathway in humans with primary DCM, we speculate that activation of CGAS is likely due to increased DSBs in the genomic DNA and the release of genomic DNA into the cytosol^[11–13]^. However, concomitant mitochondrial DNA damage and its release to the cytosol cannot be excluded. Moreover, although the COMET assay also provides robust evidence of DNA damage, the exact nature of the damaged DNA at the nucleotide resolution could not be determined. Finally, the human heart samples were not analyzed for the presence of viral DNA or RNA, reflective of viral infection, which is known to activate the CGAS-STING1 pathway^[9,10,23]^.

Increased CGAS and TBK1 axis is known to activate the expression of the pro-inflammatory cytokines^[9,10,23]^. Myocardial inflammation secondary to activation of the CGAS pathway in conjunction with the activation of the nuclear DDR pathway induces cell death, fibrosis, senescence, and cell cycle arrest and is pathogenic in DCM^[13,15]^. It is plausible that part of the myocardial inflammation that is commonly observed in cardiomyopathies and heart failure is due to activation of the inflammatory pathways in response to DNA damage, including DSBs. The pathogenic nature of activation of the CGAS pathway was recently illustrated in a mouse model whereby deletion of the *Mb21d1* gene, which encodes the CGAS protein, prolonged survival, improved cardiac function, reduce cell death, and attenuated fibrosis^[15]^.

Collectively, the findings of the present and the previous studies set the stage for testing the effects of inhibition of the DDR and CDSP pathways in DCM.
